# Supplementary material for: HDAC2- and EZH2-Mediated Histone Modifications Induce PDK1 Expression through miR-148a Downregulation in Breast Cancer Progression and Adriamycin Resistance
Source: Cancers (Basel). 2022 Jul 23;14(15):3600. doi: 10.3390/cancers14153600 (PMC9329997; doi:10.3390/cancers14153600)
Supplement: Supplementary file 1 [file cancers-14-03600-s001.zip › cancers-1791213-supplementary materials/Supplementary Figures and Table.pdf]

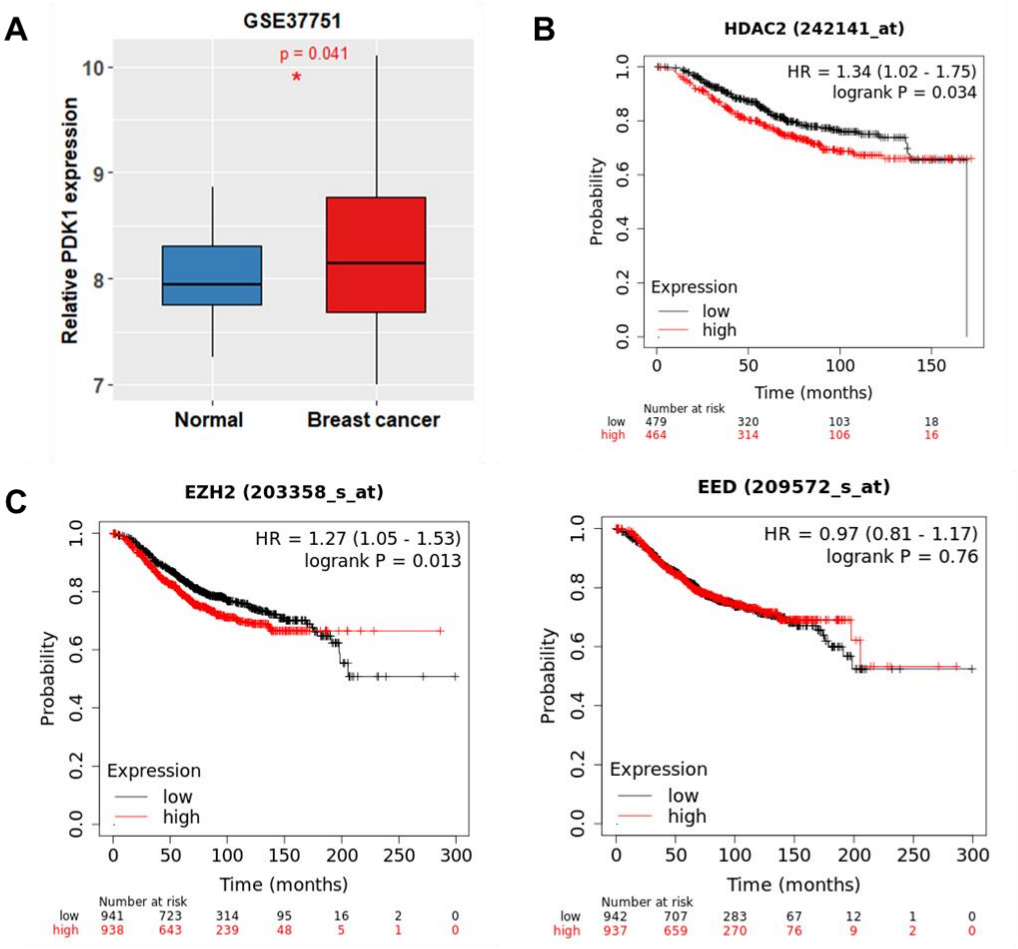

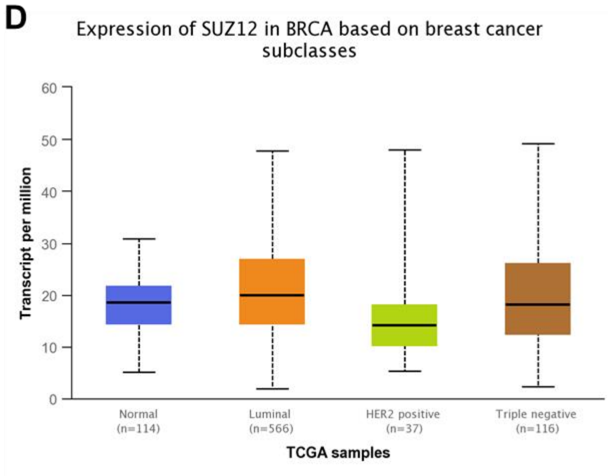

Comparison	Statistical significance
Normal-vs-Luminal	3.27E-07
Normal-vs-HER2 Positive	2.51E-01
Normal-vs-TNBC	3.60E-02
Luminal-vs-HER2 Positive	8.84E-01
Luminal-vs-TNBC	3.32E-01
HER2 Positive-vs-TNBC	8.03E-01

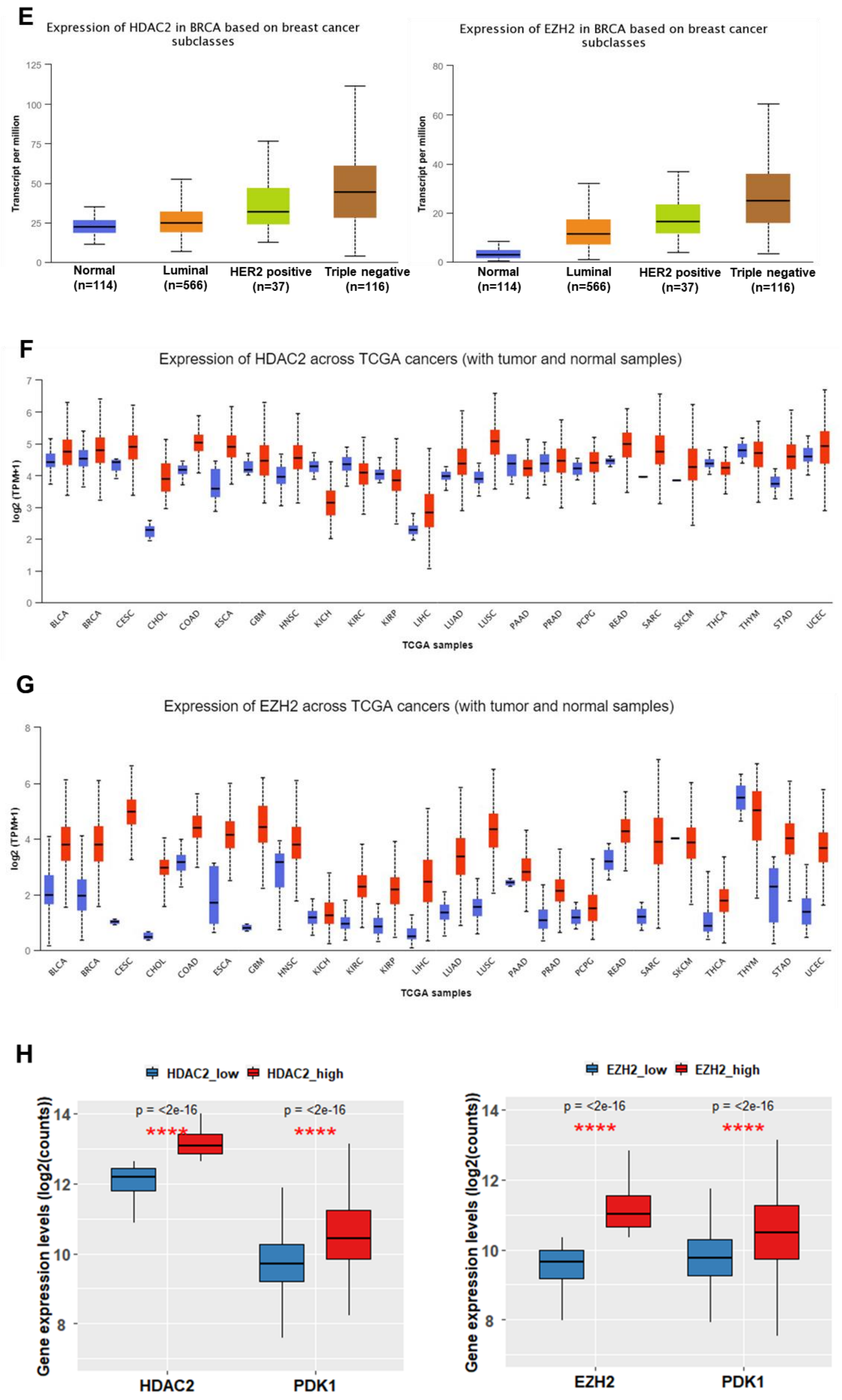

Figure S1. PDK1, HDAC2 and EZH2 are highly expressed in cancers and are associated with poor prognosis. **(A)** The expression levels of PDK1 in normal breast and breast cancer tissues were analyzed using GSE37751

dataset. **(B, C)** The overall survival (OS) probability of TCGA breast cancer samples was analyzed in groups with high- and low-expression of HDAC2 (HR = 1.34, p = 0.034), EZH2 (HR = 1.27, p = 0.013) and EED (HR = 0.97, p = 0.76) by Kaplan-Meier Plotter online analysis website. **(D, E)** The levels of SUZ12, HDAC2 and EZH2 in different molecular subtypes of breast cancer based on the TCGA breast cancer dataset. (**F, G)** The expression levels of HDAC2 and EZH2 in 24 cancer types from TCGA datasets (normal(blue) and tumor(red) samples). (**H**) The expression levels of PDK1 were analyzed in HDAC2 high and low expression groups (HDAC2_high, HDAC2_low) based on TCGA BRCA dataset. The expression levels of PDK1 were analyzed in EZH2-high and EZH2-low expression groups (EZH2_high, EZH2_low) based on TCGA BRCA dataset.

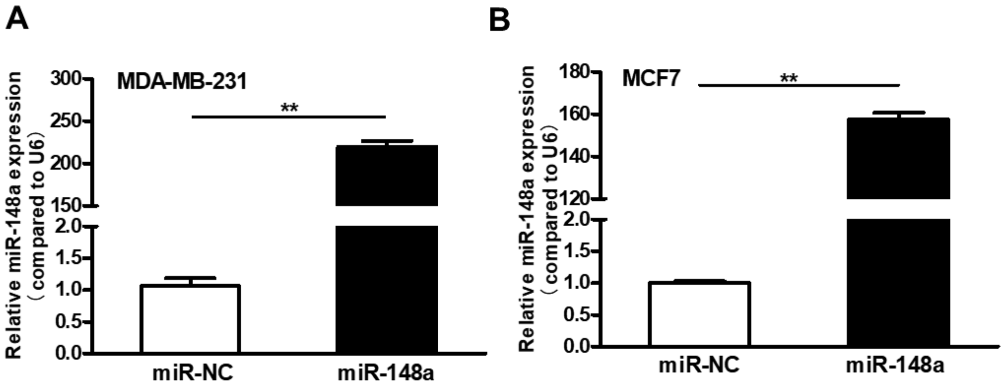

Figure S2. Construction of overexpression cell lines of miR-148a in MDA-MB-231 and MCF7. **(A)** The expression levels of miR-148a in MDA-MB-231 stable overexpression cell lines (MDA-MB-231 miR-NC and MDA-MB-231 miR-148a). (**B)** The expression levels of miR-148a in MCF7 stable overexpression cell lines (MCF7 miR-NC and MCF7 miR-148a). (U6 was used as an endogenous control, and ** indicates $p < 0.01$).

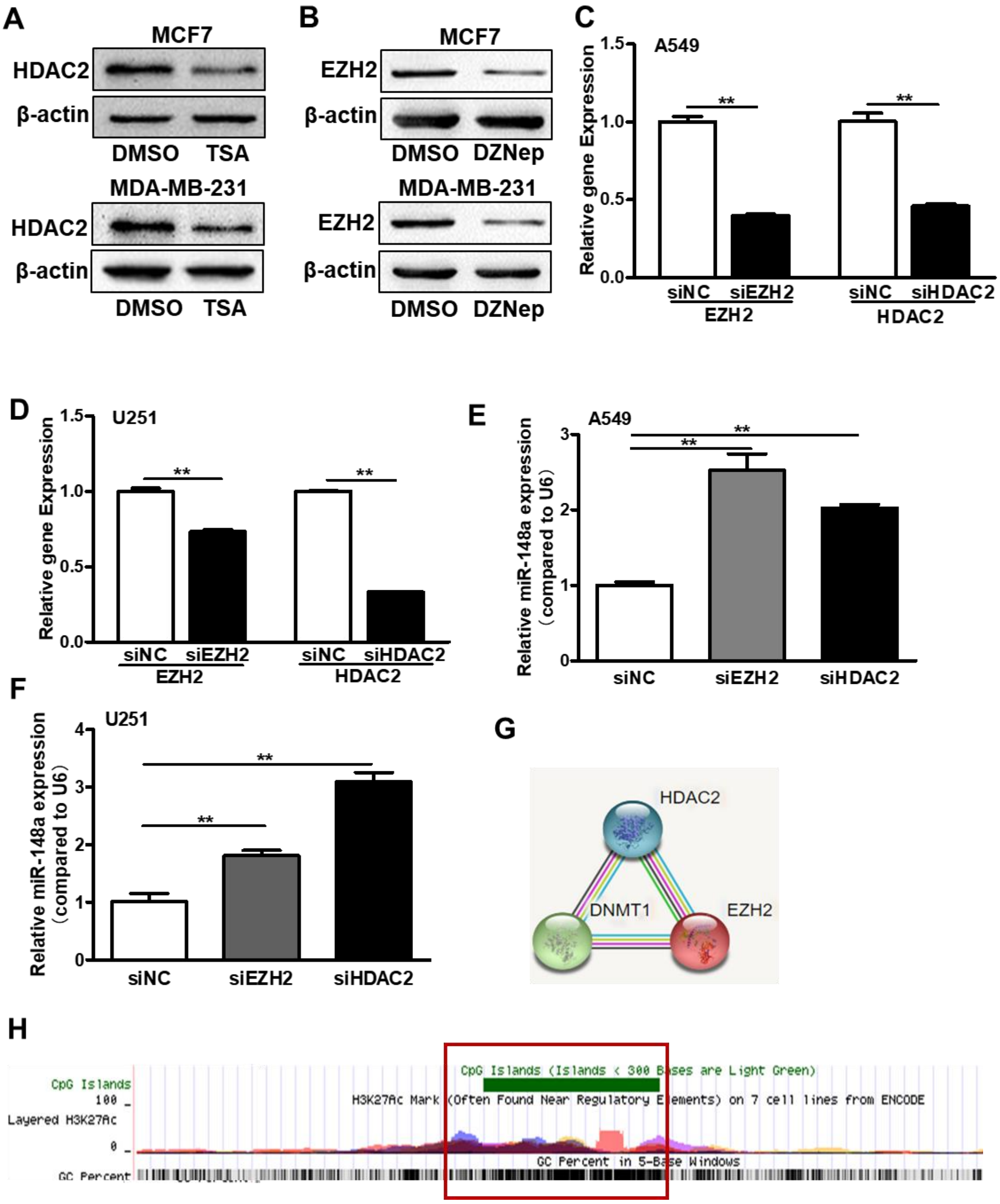

Figure S3. The expression of miR-148a was up-regulated in knocked down EZH2 or HDAC2 cells. **(A)** The expression levels of HDAC2 in MCF7 and MDA-MB-231 cells treated with DMSO and TSA. (**B)** The expression levels of EZH2 in MCF7 and MDA-MB-231 cells treated with DMSO and DZNep. (**C, D)** The qRT-PCR assay was used to measure expression levels of EZH2 and HDAC2 in A549 and U251 cells treated with siNC, siEZH2, or siHDAC2. (**E, F)** The expression levels of miR-148a in A549 and U251 cells treated with siNC, siEZH2, or siHDAC2. (**G)** The correlation analysis between HDAC2, EZH2, and DNMT1 was performed using STRING v11.5, an online analysis website. The more connecting lines between genes, the higher the correlation. (**H)** The CpG islands and histone modification (H3K27AC) regions on the upstream of the miR-148a promoter. Red box indicates the overlap region.

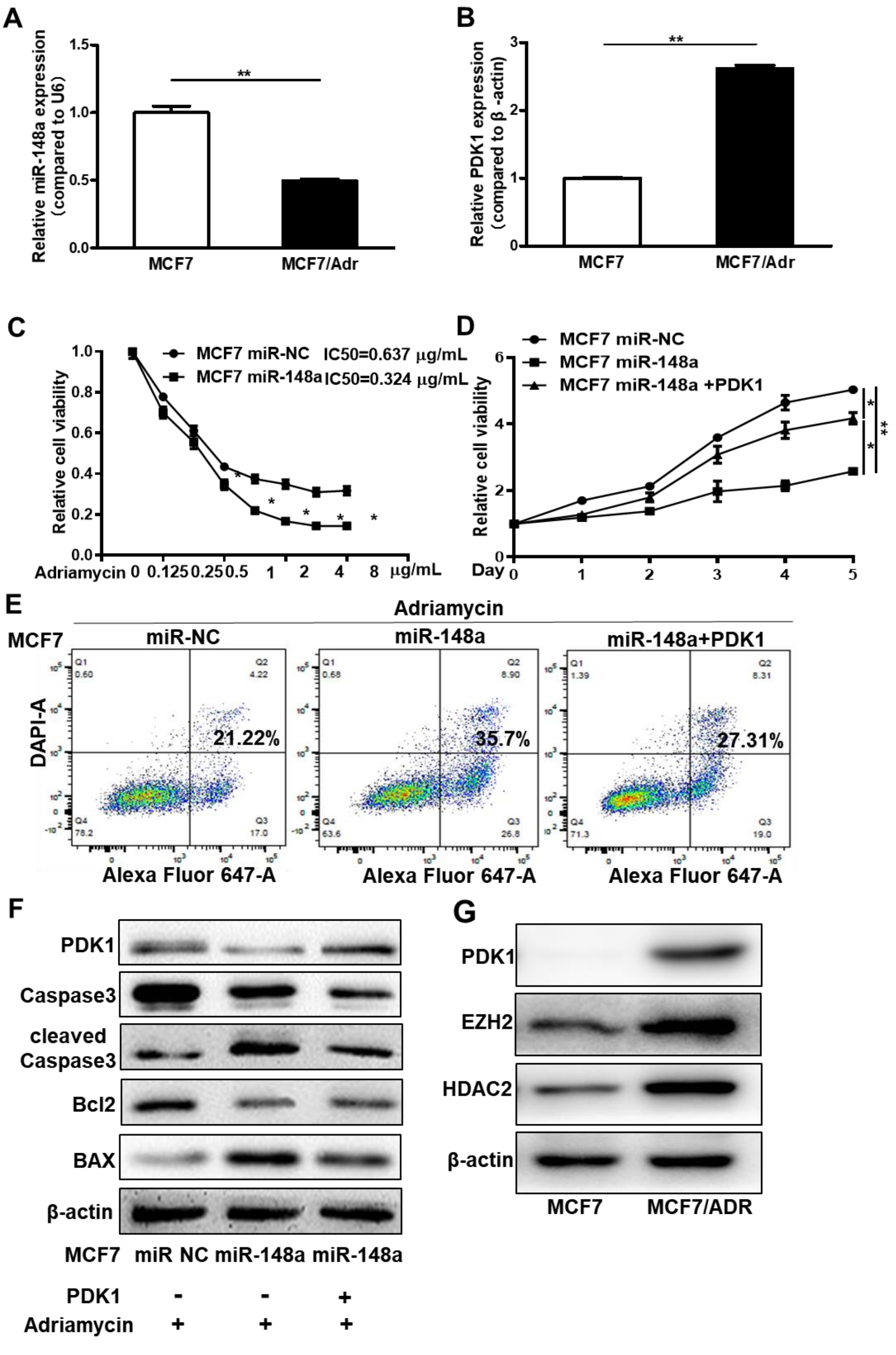

Figure S4. MiR-148a/PDK1 regulated Adriamycin resistance by controlling cell apoptosis**.** **(A, B)** The expression levels of miR-148a and PDK1 in MCF7 and MCF7/ADR (Adriamycin-resistant breast cancer cell line) cells. (**C)** MCF7 cells carrying miR-NC and miR-148a were treated with Adriamycin (0-8μg/ml), and the IC50 values were calculated by CCK8 assays. (**D)** The proliferative abilities of MCF7 miR-NC and miR-148a overexpression groups treated with 0.15 μg/ml Adriamycin were detected by CCK8 assays. (**E)** Flow cytometry was used to detect the apoptosis levels in MCF7 cells carrying miR-NC and miR-148a. (**F)** The protein expression levels of BAX, Bcl-2, cleaved Caspase3, Caspase3, and PDK1 were detected by Western blotting in MCF7 cells treated with 0.15 μg/ml Adriamycin. (**G)** The expression levels of PDK1, EZH2, and HDAC2 were detected in MCF7 and MCF7/ADR cells. (*Indicates $p < 0.05$, and ** indicates $p < 0.01$).

Table S1. The patient information of breast cancer tissues used in this study.

Patient ID	*Age*	*Histological grade*	*ER*	*PR*	*HER2*	*Lymphangion-invasion*
1 P/C	53	III	−	+	+	+
2 P/C	54	II	−	−	−	+
3 P/C	46	II	−	−	+	−
4 P/C	65	III	+	+	+	−
5 P/C	54	II	−	−	+	+
6 P/C	72	II	+	−	−	+
7 P/C	38	II	+	+	+	+
8 P/C	52	II	−	−	−	+
9 P/C	62	II	+	+	NA	−
10 P/C	63	II	+	−	+	−
11 P/C	66	II	+	+	+	−
12 P/C	59	III	−	−	+	+
13 P/C	51	III	+	−	+	−
14 P/C	52	II	+	+	+	−
15 P/C	41	II	+	+	+	−
16 P/C	60	II	+	+	+	+
17 P/C	60	II	+	+	+	NA
18 P/C	51	III	+	+	+	−
19 P/C	44	II	+	−	+	+
20 P/C	43	II	+	+	+	−
